# Smoking during pregnancy: Childbirth and Health Study in Primary Care in Iceland

**DOI:** 10.3109/02813432.2013.869409

**Published:** 2014-03

**Authors:** Asthildur Erlingsdottir, Emil L. Sigurdsson, Jon Steinar Jonsson, Hildur Kristjansdottir, Johann A. Sigurdsson

**Affiliations:** ^1^Department of Family Medicine, University of Iceland, Gardabaer, Iceland; ^2^Gardabaer Health Care Centre, Gardabaer, Iceland; ^3^Solvangur Health Care Centre, Hafnarfjordur, Iceland; ^4^Department of Midwifery, Faculty of Nursing, University of Iceland; ^5^Directorate of Health, Reykjavik, Iceland; ^6^General Practice Research Unit, Department of Public Health and General Practice, Norwegian University of Science and Technology (NTNU), Trondheim, Norway

**Keywords:** Antenatal care, childbirth and health, general practice, Iceland, pregnancy, primary health care, smoking habits

## Abstract

*Objective*. To study the prevalence and possible predictors for smoking during pregnancy in Iceland. *Design.* A cross-sectional study. *Setting.* Twenty-six primary health care centres in Iceland 2009–2010. *Subjects.* Women attending antenatal care in the 11th–16th week of pregnancy were invited to participate by convenient consecutive manner, stratified according to residency. A total of 1111 women provided data in this first phase of the cohort study. *Main outcome measures.* Smoking habits before and during early pregnancy were assessed with a postal questionnaire, which also included questions about socio-demographic background, physical and emotional well-being, and use of medications. *Results.* The prevalence of smoking prior to pregnancy was 20% (223/1111). During early pregnancy, it was 5% (53/1111). In comparison with women who stopped smoking during early pregnancy, those who continued to smoke had on average a significantly lower level of education, had smoked more cigarettes per day before pregnancy, and were more likely to use nicotine replacement therapy in addition to smoking during pregnancy. A higher number of cigarettes consumed per day before pregnancy and a lower level of education were the strongest predictors for continued smoking during pregnancy. *Conclusion.* The majority of Icelandic women who smoke stop when they become pregnant, and the prevalence of smoking during pregnancy in Iceland is still about 5%. Our results indicate stronger nicotine dependence in women who do not stop smoking during pregnancy. Awareness of this can help general practitioners (GPs) and others providing antenatal care to approach these women with more insight and empathy, which might theoretically help them to quit.

Smoking during pregnancy is a major preventable risk factor for foetal and neonatal morbidity and mortality.

- This study shows that the prevalence of smoking during early pregnancy in Iceland is 5%.
- A history of heavy smoking before pregnancy and low level of education characterize the women who do not stop smoking early in pregnancy.
- Our results can help GPs and midwives to identify women at risk of continued smoking during pregnancy and possibly assist them to quit.

## Introduction

Smoking during pregnancy is considered a major preventable risk factor for foetal and neonatal morbidity and mortality [1]. Smoking during pregnancy increases the risk of preterm delivery, low birth weight, and many other adverse pregnancy outcomes, and smoking cessation early in pregnancy has been shown to decrease these risks [2–4].

About half of women who smoke before becoming pregnant stop during pregnancy [5,6]. Previous studies have shown that the proportion of women who smoke throughout pregnancy is 7–17% in high-income countries like Sweden, Norway, Germany, Canada, the USA, and Australia [2,7–11]. Most women who stop do so in the first trimester without any treatment [5]. Interventions to promote smoking cessation during pregnancy have not proved to be very effective. In a large meta-analysis, Lumley et al. concluded that the overall success of interventions was a 6% increase in smoking cessation during pregnancy, with the most effective intervention being to provide incentives, which increased the cessation rate during pregnancy by 24%. Other methods, including cognitive behavioral therapy (CBT), nicotine-replacement therapy (NRT), and “stages of change” theory strategies each appeared to increase the cessation rate by only 6% [12].

Younger women [6,9,13,14], multiparous women [13–15], women of lower socioeconomic and educational status [6,8,13], and women who are not cohabiting with the father [8,13,14] have been found more likely than others to smoke throughout pregnancy. Women continuing to smoke while pregnant are more likely than those who quit to have depressive symptoms [16], and to have experienced stressful life events [6,8]. Preconception smoking intensity [6,14] and passive smoking during pregnancy also correlate positively with smoking during pregnancy [8,13].

Midwives and general practitioners in primary care are responsible for antenatal care in uncomplicated pregnancies in Iceland and many other countries [17,18]. It is therefore their task to identify women at risk of smoking during pregnancy in an effort to help them to stop smoking, for their own benefit and that of their baby.

Recent studies on the aforementioned risk factors are lacking in the Nordic countries. The aim of this study was therefore to investigate the prevalence and possible predictors for smoking during pregnancy in Iceland.

## Material and methods

### Demography and organisational structure of healthcare service

The size of the Icelandic population is 320,000, 70% of which lives in and around the capital city of Reykjavik. Primary healthcare is carried out at 45 healthcare centres, staffed by general practitioners (GPs), nurses, midwives, and other ancillary staff. Obstetricians are working part-time at the larger healthcare centres. The health care providers at the centres are responsible for the antenatal and well-baby care in Iceland. They work closely with the obstetric units at the nearest hospital, where more specialized care is provided for women who are considered to be at high risk during pregnancy. The status of healthcare is similar to that of the other Nordic countries [19].

### Design

This study is a part of the Childbirth and Health Study in Primary Care in Iceland, which has been described in more detail elsewhere [20]. Its design and questionnaires were based on a similar cohort study on pregnant women carried out in Sweden in 1999–2000, *Kvinnors upplevelse av barnafödande* (the “KUB” study) [21]. The Childbirth and Health Study is a population-based cross-sectional and cohort study of pregnant women, who answered postal questionnaires around the 16th week of pregnancy (Phase I), at 5–6 months after childbirth (Phase II), and 18–24 months after delivery (Phase III). The women answered a comprehensive questionnaire about socio-demographic and obstetric background, physical and emotional well-being, use of medications, social support, and expectations about antenatal and delivery care. The women were asked about their smoking habits before and during pregnancy (yes or no) and, if yes, how many cigarettes per day they smoked. We used the Edinburgh Postnatal Depression Scale (EPDS) questionnaire to identify depressive symptoms with a score cut-off point > 15. Convenient consecutive sampling was used for phase I, stratified according to residency, to attain a distribution similar to the distribution in the entire country, with the ratio 70:30 for urban and rural residency, respectively. Education was defined as follows: elementary school covering 10 years; college or similar covering 3–4 years after elementary school; technical education or university less than four years; university more than four years.

### Study population

The first phase (Phase I) was carried out over a period of 12 months, from February 2009 to March 2010. A total of 26 healthcare centres (out of 45) were selected, distributed evenly around the country, with a catchment area covering around 60% of all maternity care provided by primary care, counting around 3000 pregnancies per year. Consecutively, midwives providing antenatal care informed women 18 years and older of the study on their first antenatal visit and invited them to participate with the aim of reaching around 1500 participants. One week later the questionnaire was sent to those who responded positively to the first invitation. One letter of reminder was sent to all participants.

A total of 1111 of 1765 pregnant women invited (63%) filled out the questionnaire, thus participating in Phase I. This constituted approximately 23% of all pregnant women in Iceland in 2009. In this part of our study, we will refer only to the first phase.

### Statistical analysis

Descriptive data are presented as mean values with standard deviations and percentages. Statistical significance was deemed to be at p-values of less than 0.05, with a two-sided significance test. A Pearson's chi-squared test was used for assessing significance between groups, and a t-test was used to check for difference in significance between continuous variables. A logistic regression analysis was done, with current smoking as the dependent variable, and we used several variables as independent variables, beginning with those significant in Table II. In the end the model with marital status, education, number of cigarettes smoked prior to pregnancy, and the Edinburgh Postnatal Depression Scale (EPDS) as independent variables was used. A normality assumptions test was done before running the regression analysis. The statistical software package IBM, SPSS version 20, was used for all data analysis.

**Table I.** Characteristics of participants in the Childbirth and Health Study 2009–2010 (percentages and absolute figures in parentheses or as otherwise described): Similar variables are shown for the entire birth cohort in Iceland in 2009.^1^

	Participants (n = 1111)	Iceland (n = 4939^2^)
Parity:		
Primipara	40 (439)	40 (2005)
Multipara	60 (671)	60 (3021)
Age:		
18–19	2 (18)	3 (185)
20–24	15 (168)	17 (841)
25–29	36 (405)	34 (1691)
30–34	30 (328)	29 (1434)
35–39	14 (155)	14 (722)
> 40	3 (37)	3 (168)
Mean age (yrs)	29.4	29.7
Education:		
Elementary	11 (123)	28^3^ (18 000)
High school or similar	26 (291)	36^3^ (23 100)
Technical education or university < 4 years	26 (291)	X^4^
University > 4 years	36 (404)	36^3,5^ (23 100)
Combined higher education^6^	89 (986)	72^3^ (46 200)
Residence:		
Capital area	69 (763)	67 (3378)
Rural area	31 (347)	33 (1648)
Marital status:		
Married/cohabiting	93 (1032)	84 (4241)
Single	3 (31)	X^4^
Other	4 (48)	16 (785)

Notes: ^1^Statistics Iceland, http://www.hagstofa.is (last checked on 2 April 2013). ^2^All deliveries in Iceland in 2009 (Icelandic Birth Register for 2009). ^3^Figures for pregnant women not available, but only for the total sample (n = 64 000) of all Icelandic women aged 20–49 years. ^4^No figures for comparison available. ^5^Counts all university education regardless of duration. ^6^All education after elementary school.

**Table II.** Characteristics of women who quit smoking while pregnant and those who smoke during early pregnancy (means and standard deviation [SD] in parentheses, and percentage and absolute figures within parentheses respectively).

	Stopped smoking during pregnancy (n = 170)	Smoked during early pregnancy (n = 53)	p-values
Number of cigarettes per day before pregnancy; mean (SD)	8 (5.3)	15 (5.2)	< 0.001
Mean age, years (SD)	28 (4.8)	28 (6.0)	0.466
Multiparous	48 (82/170)	60 (32/53)	0.123
Marital status:			0.473
Married/co-habiting	84 (142/170)	79 (42/53)	
Single	17 (28/170)	21 (11/53)	
Education:			< 0.001
Elementary school	20 (33/169)	42 (22/53)	
Higher education	81 (136/169)	59 (31/53)	
Employment status:			0.065
Receiving welfare	9 (15/170)	19 (10/53)	
Working	71 (120/170)	55 (29/53)	
Housewife	1 (2/170)	4 (2/53)	
Student	17 (28/170)	15 (8/53)	
Other	3 (5/170)	8 (4/53)	
Use of medications during pregnancy:			
Nicotine replacement therapy	3 (4/163)	21 (10/47)	< 0.001
Psychotropic medications^1^	7 (11/163)	14 (7/49)	0.097
Alcohol consumption during pregnancy	2 (3/170)	2 (1/53)	0.953
Alcohol consumption prior to pregnancy:			0.133
Rarely	55 (93/170)	68 (36/53)	
Once a week or less	35 (60/170)	23 (12/53)	
More than once a week	7 (11/170)	9 (5/53)	
Never	4 (6/170)	0	
Major life events last 12 months^2^	42 (71/170)	34 (18/53)	0.311
Depressive symptoms^3^	7 (12/169)	11 (6/53)	0.326
Own perceived health:			0.423
Excellent/very good	79 (135/170)	74 (39/53)	
Fair	17 (29/170)	25 (13/53)	
Poor/very poor	4 (6/170)	2 (1/53)	
Planned pregnancy:			0.312
Yes	56 (94/168)	45 (24/53)	
No, but welcome	31 (52/168)	43 (23/53)	
No, the timing could have been better	8 (14/168)	9 (5/53)	
No, I considered termination of the pregnancy	5 (8/168)	2 (1/53)	
Information about harm of smoking in pregnancy:			0.097
None/not enough	17 (28/167)	8 (4/53)	
Enough/too much	83 (139/167)	93 (49/53)	

Notes: ^1^Psychotropic medications: hypnotic, antidepressant, or sedative medications. ^2^Major life events: ≥ 2 of the following life events: having been in a serious accident or had serious illness, serious illness, accident to or death of a family member, serious concerns about a family member, divorce or separation, forced to move household or change jobs, made redundant, had feelings of insecurity at work, serious financial problems, been legally prosecuted. ^3^Edinburgh Postnatal Depression Scale (EPDS) ≥ 15.

### Ethical considerations

The study was approved by the National Bioethical Committee in Iceland (VSNb2008010023/03-1) and reported to the Data Protection Authority (S3695/2008 LSL/). The study was also approved by the professional authorities of the healthcare centres approached.

## Results

The prevalence of smoking prior to pregnancy was 20% (223/1111) and 5% (53/1111) during early pregnancy in the study sample. Of those who smoked before becoming pregnant, 76% (170/223) had stopped smoking at 16 weeks, but 24% (53/223) continued smoking during pregnancy.

Table I gives the participants’ background characteristics. For comparison, similar data are shown for all pregnant women in 2009 in Iceland. Table II illustrates the prevalence of various demographic, socioeconomic, and health-related characteristics potentially associated with smoking in the group of women who stopped smoking during pregnancy and the group that continued smoking during pregnancy. As shown, women in the group that continued to smoke had on average a significantly lower level of education, had on average smoked more cigarettes per day before pregnancy, and were more likely to use nicotine replacement therapy during pregnancy than women in the group that stopped smoking (Table II).

Before pregnancy 45% (55/123) of women who had elementary education only smoked, and 40% (22/55) of them were still smoking at the 16th week of pregnancy. Among women with higher education, the prevalence of smoking was 17% (167/984) before pregnancy and 19% (31/167) of them were still smoking at the 16th week of pregnancy.

The number of cigarettes consumed per day before pregnancy and a lower level of education were the strongest predictors for continued smoking during pregnancy (Table III). The predictability of the model was 80.8% and the variance measured as Nagelkerke R-square was 0.335.

**Table III.** Factors predictive of continued smoking in pregnancy: Logistic regression analysis.

	Adjusted OR (95% CI)^1^	p-values	B
Lower level of education	2.5 (1.2–5.5)	0.021	0.918
Increased number of cigarettes smoked per day before pregnancy	1.2 (1.1–1.3)	< 0.001	0.201
Married	0.8 (0.3–2.1)	0.664	− 0.213
Depressive symptoms^2^	1.6 (0.4–6.1)	0.474	0.482

Notes: ^1^95% confidence interval. ^2^Edinburgh Postnatal Depression Scale (EPDS) ≥ 15.

## Discussion

This comprehensive study on pregnancy and antenatal care carried out in the primary care setting in Iceland shows that the prevalence of smoking among women prior to pregnancy was around 20% and dropped to 5% in early pregnancy. A lower level of education and intensive nicotine dependence seem to be the main predictors of smoking in this group.

The main strength of this study is the size of the study sample; it included 23% of all pregnant women in Iceland. The study sample is broadly representative of the general pregnant population in Iceland. Most of the pregnant women in our sample were between 24 and 34 years old, which means a potentially longer period for higher education in our study group. National data on the educational level of pregnant women were not available. Our classification of education was furthermore not quite comparable to the national statistics. Therefore, comparison between our data and the national data on education requires caution. However, women with a higher level of education seem to be overrepresented in our study [20]. Since education is one of the factors predictive of smoking, in both this and other studies [6,8,13], the prevalence of smoking among pregnant women in Iceland might be higher than our results suggest.

The study has some limitations. The questionnaire focused more on attitudes and expectations about pregnancy and delivery than comprehensive questions regarding smoking itself. For example, questions on passive smoking in a social or work environment were not included. Furthermore, the study was carried out in the beginning of the second trimester, so we do not have information on women who may have stopped smoking later in pregnancy or those who may have relapsed before giving birth.

In our study, a lower level of education was the only demographic factor that was significantly more common amongst women who continued to smoke during pregnancy than amongst those who did stop. This result is in accordance with results of previous studies [6,8,13]. There was, however, no difference in other factors that have previously been shown to characterize pregnant smokers, such as marital status, age, socio-economic status, or parity [6,8,9,13–15].

In our study factors associated with stress or symptoms of depression, such as the EPDS, an unplanned pregnancy, or poor self-reported health, did not have any predictive value for continued smoking during pregnancy.

Smoking more cigarettes per day before becoming pregnant was predictive of continued smoking during pregnancy (OR = 1.22), and the use of nicotine replacement therapy during pregnancy was much more common among the women continuing to smoke during pregnancy, than among those who stopped (21% and 3%, respectively, p = ˂0.001). These results suggest that nicotine dependence is the main characteristic of women who continue to smoke during pregnancy. Indicators of other substance dependence, such as alcohol consumption before and during pregnancy and the use of addictive medication, were not predictive of smoking during pregnancy in this study. The incidence of this behaviour was low, which is probably explained by the fact that known substance abusers do not normally participate in antenatal care in the primary care setting in Iceland, but are referred to specialized care. Smoking in this group is likely to be higher than in the general pregnant population [22].

The main conclusion of this study is that the majority of Icelandic women who smoke stop when they become pregnant, and that the prevalence of smoking during pregnancy in Iceland is still about 5%. The women who do continue smoking in early pregnancy have on average a lower level of education, and they smoke on average more before pregnancy, which could indicate a more intensive nicotine dependence than women who are able to stop smoking upon becoming pregnant. Since the safety and efficacy of medical treatment for smoking during pregnancy is not well established [23], and the efficacy of widely used smoking therapies, such as CBT and the “stages of change” theory strategies, is also questionable, GPs and others providing antenatal care to assist pregnant women who smoke to stop clearly face a big challenge. One way of dealing with nicotine addiction in pregnancy could be to provide these women with support with more insight and empathy [24].
